# Landscape of Savolitinib Development for the Treatment of Non-Small Cell Lung Cancer with MET Alteration—A Narrative Review

**DOI:** 10.3390/cancers14246122

**Published:** 2022-12-12

**Authors:** Xiaokuan Zhu, Yao Lu, Shun Lu

**Affiliations:** 1 Department of Shanghai Lung Cancer Center, Shanghai Chest Hospital, Shanghai Jiao Tong University, Shanghai 200030, China; 2 AstraZeneca China, Shanghai 201200, China

**Keywords:** savolitinib, non-small cell lung cancer, *MET* aberrations, *EGFR*, tyrosine kinase inhibitor

## Abstract

**Simple Summary:**

In this article, we outline updates on the clinical development of savolitinib, a novel, reversible c-MET kinase inhibitor conditionally approved in China for treatment of advanced non-small cell lung cancer (NSCLC) patients harboring *MET* exon 14 skipping mutation (*MET*ex14). Savolitinib was developed as a monotherapy for NSCLC with *MET* alterations, and in combination with epidermal growth factor receptor (*EGFR*) inhibitors for patients who developed resistance to EGFR–TKIs because of *MET* alterations. Savolitinib showed anti-tumor activity in preclinical models. The early phase I trial established the recommended phase II dose to be 600 mg once-daily. Savolitinib plus osimertinib showed beneficial efficacy and safety in *EGFR* mutant patients with acquired resistance due to *MET* amplification and/or c-MET overexpression. Benefits were noted with savolitinib in Chinese patients with pulmonary sarcomatoid carcinoma and other NSCLC subtypes positive for *MET*ex14 mutation. Results from phase III trials are awaited to further confirm the beneficial effects from early phase trials.

**Abstract:**

Non-small cell lung cancer (NSCLC) is increasingly being treated with targeted therapies. Savolitinib (Orpathys^®^) is highly selective mesenchymal epithelial transition (MET)–tyrosine kinase inhibitor (TKI), which is conditionally approved in China for advanced NSCLC with *MET* exon 14 skipping mutations (*MET*ex14). This article summarizes the clinical development of savolitinib, as a monotherapy in NSCLC with *MET*ex14 mutation and in combination with epidermal growth factor receptor (*EGFR*) inhibitor in post EGFR–TKI resistance NSCLC due to *MET*-based acquired resistance. Preclinical models demonstrated anti-tumor activities in *MET*-driven cancer cell line and xenograft tumor models. The Phase Ia/Ib study established an optimized, recommended phase II dose in Chinese NSCLC patients, while TATTON study of savolitinib plus osimertinib in patients with *EGFR* mutant, *MET*-amplified and TKI-progressed NSCLC showed beneficial efficacy with acceptable safety profile. In a pivotal phase II study, Chinese patients with pulmonary sarcomatoid carcinoma, brain metastasis and other NSCLC subtype positive for *MET*ex14 mutation showed notable responses and acceptable safety profile with savolitinib. Currently, results from ongoing clinical trials are eagerly anticipated to confirm the efficacious and safety benefits of savolitinib as monotherapy and in combination with EGFR–TKI in acquired resistance setting in advanced NSCLC and its subtypes with *MET* alterations.

## 1. Introduction

Non-small cell lung cancer (NSCLC) accounts for approximately 80% of all lung cancers with a low 5-year survival rate of about 22% [1,2]. Most NSCLC are usually diagnosed at an advanced stage with traditional chemotherapy and radiotherapy showing limited efficacy. However, recent advances in immune therapy and targeted therapy have radically improved the treatment paradigm of NSCLC over the past decade [2]. Molecular profiling of lung cancer samples for activated oncogenes, including epidermal growth factor receptor (*EGFR*), anaplastic lymphoma kinase (*ALK*) and c–ros oncogene 1 (*ROS1*), is considered as standard-of care to select the most appropriate up-front treatment [3]. However, the identification of new therapeutic targets remains a high priority. Recently, mesenchymal-epithelial transition (*MET*) exon 14 skipping mutations (*MET*ex14) and high-level *MET* amplification have emerged as one of the novel, actionable oncogenic alterations in NSCLC, sensitive to MET inhibitors [4,5].

**Figure 1:**
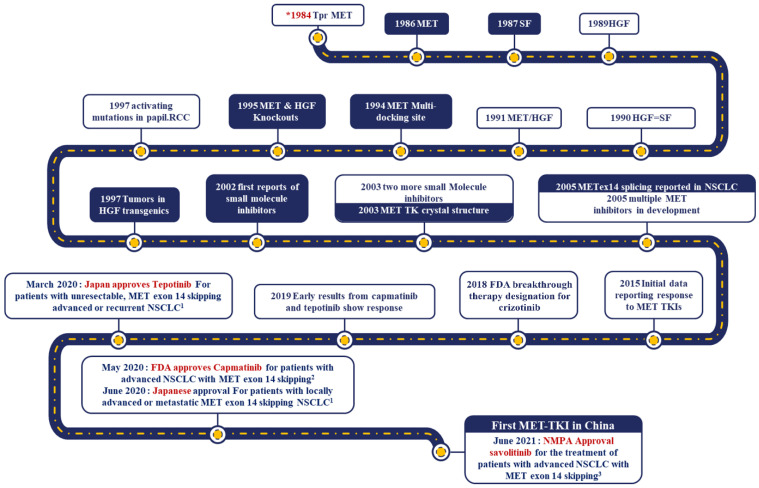
Exploration of *MET* as oncogene and the journey leading to the development of MET–TKI. ^1^ Based on overall NSCLC population. ^2^ Based on treatment-naïve NSCLC population. ^3^ Based on *EGFR*-positive treatment-naïve NSCLC population.

MET is a receptor tyrosine kinase activated by binding ligand hepatocyte growth factor (HGF) which plays a key physiological role in the interaction between mesenchyme and epithelia during embryonic wound closure and embryogenesis [6,7,8,9]. At cellular levels, MET-TK activity transduces mitogenesis by activating Ras–Raf–MAPK signaling pathway and motogenic signals by activating phosphoinositide 3–kinase (PI–3K) pathway upon HGF binding [10]. Aberrant MET/HGF signaling promotes mitogenesis, invasion and angiogenesis, thus contributing towards tumorigenesis and progression of cancer [11]. Importantly, significant implications for tumorigenesis are observed due to crosstalk between downstream signal pathways of *MET* and *EGFR* [12]. The oncogenic role of *MET* was first discovered in 1984 as a part of an oncogenic fusion with the translocated promoter region gene in a mutagenized osteosarcoma cell line [13]. *MET* alterations, including amplification, mutations, gene fusion, MET/HGF protein over expression and the crosstalk between dysregulated *MET* and other signaling pathways, are associated with poor prognosis in cancers, and thus, molecularly targeted [4]. *MET*ex14 mutations are the most commonly reported oncogenic mutations. Exon 14 encodes the 47-amino acid juxtamembrane domain of the *MET* receptor, a key regulatory region that prevents *MET* over signaling. *MET*ex14 mutations include a heterogeneous group of mutations with base substitutions or indels that disrupt the branch point of intron 13, the 3′ splice site of intron 13 or the 5′ splice site of intron 14, producing a *MET* variant that lacks the exon 14 leading to disruption of cellular signaling [14]. The identification of *MET* oncogene and the journey leading to development of MET–TKIs is represented in Figure 1.

Clinical studies conducted earlier suggest that activation of *MET* can act as primary oncogenic driver, or secondary driver of acquired resistance to targeted therapy in subsets of lung cancer [9,10,11]. *MET*ex14 mutations occur in approximately 0.9 to 4% of NSCLC cases across all histologic subtypes [6] and are enriched in pulmonary sarcomatoid carcinoma (PSC) (20 to 31%), a rare subtype of poorly differentiated NSCLC [15,16]. Furthermore, 1 to 5% of NSCLC harbors de novo *MET* gene amplification, while 15% of cases in *MET*ex14-mutated NSCLC report *MET* amplification [17,18]. *MET* fusion is known to occur in 0.5% [18] and MET protein overexpression in 13.7 to 63.7% of NSCLC patients [17]. Significant cross talk between aberrant *MET* pathway and other signaling pathways, especially *EGFR* results in acquired resistance to EGFR tyrosine kinase inhibitors (TKIs) in patients with NSCLC [19]. Mechanistically, *MET* amplification causes EGFR–TKI resistance by activating *EGFR*-independent phosphorylation of *ErbB*3 and downstream activation of the PI3K/AKT pathway, providing a bypass pathway in the presence of an *EGFR* inhibitor [20]. Thus, concomitant inhibition of both *EGFR* and *MET* would be required to overcome resistance to *EGFR* inhibitors by *MET* amplification [19]. Approximately, 5–22% of NSCLC patients with first- or second-generation EGFR–TKI resistance [18,21] and 5–50% patients with third generation EGFR–TKI resistance harbor *MET* amplification [22], while *MET* amplification as a co-driver occurs in 2–11% *EGFR*-positive treatment-naïve NSCLC patients [23,24]. The incidence of high MET expression after EGFR–TKI resistance is as high as 30.4 to 37% [25]. The proportion of different *MET* alterations in NSCLC patients is summarized in Table 1.

Currently the FDA approved MET–TKIs are capmatinib and tepotinib with crizotinib granted as breakthrough therapy designation, while savolitinib is conditionally approved in China [26,27,28]. Further, for *EGFR*-mutated NSCLC with *MET* amplification treatment, efficacy of combination of MET–TKIs with EGFR–TKIs has been preliminarily approved by several clinical trials [29,30]. The clinical development strategy for savolitinib is centered both as monotherapy for advanced *MET*ex14-altered NSCLC and in combination with EGFR–TKI for correction of *MET*-driven acquired resistance to EGFR–TKIs [31]. In this review, we briefly describe the major milestones achieved in the clinical development of savolitinib as standard of care for NSCLC with *MET*ex14 mutation and potential treatment for NSCLC with other *MET* alterations.

## 2. Savolitinib, in Brief

**Figure 2:**
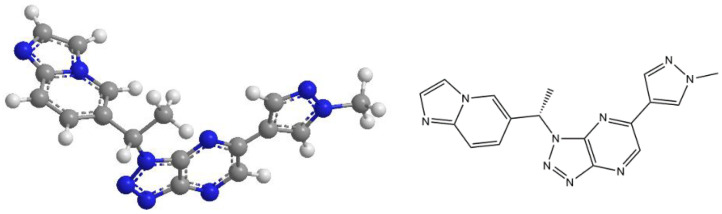
Chemical structure of Savolitinib.

Savolitinib (Orpathys^®^) is an orally bioavailable and highly selective small molecule MET–TKI that has demonstrated profound efficacy in preclinical and clinical studies of various cancers, including NSCLC, papillary renal cell carcinoma (PRCC) and gastric carcinoma [32,33,34]. Figure 2 demonstrates the chemical structure of savolitinib.

Early on, in vitro studies have established inhibitory effect of savolitinib on growth of gastric cells lines, while in vivo studies observed anti-tumor activity in human xenograft tumor models of *MET*-amplified gastric cancer and PRCC [33,34,35]. Another study by Jones and colleagues related to pharmacokinetic-pharmacodynamic (PK-PD) model observed inhibition of phosphorylated-MET by savolitinib at an effective concentration (EC)50 of 0.35 ng/mL and EC90 of 3.2 ng/mL in a cell line-derived xenograft (CDX) mice model using human lung cancer (EBC-1) and gastric cancer (MKN-45) cells [36]. Furthermore, PK studies in healthy male Chinese volunteers administered with single oral savolitinib doses of 200, 400 and 600 mg following an overnight fast or a high-fat and high-calorie breakfast prior to dosing showed no clinically relevant impact on PK and bioavailability of savolitinib [37].

**Figure 3:**
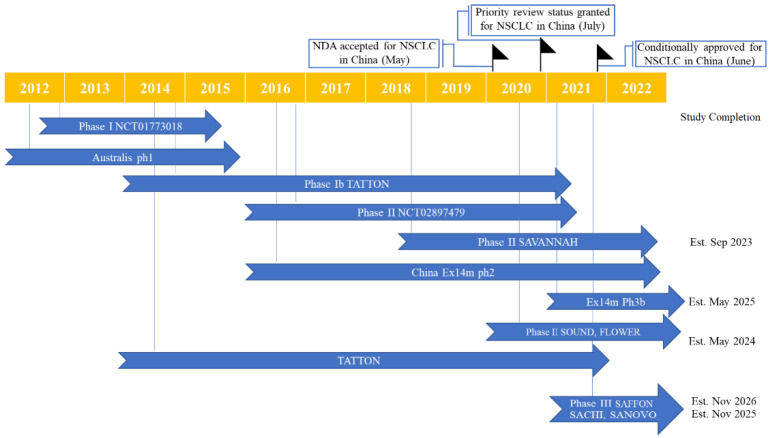
Key milestones and clinical trials in the development of savolitinib for non-small cell lung cancer.

In NSCLC with *MET* aberrations, several clinical trials have shown the potential benefit of savolitinib as a monotherapy and in combination with EGFR–TKI [30]. Savolitinib received its first approval by The National Medical Products Administration (NMPA), China for patients with *MET*ex14-altered locally advanced or metastatic NSCLC with disease-progression following systemic treatment or unable to receive chemotherapy [28]. The approval was based on a phase II trial conducted in China in patients with *MET*ex14-altered NSCLC, including patients with the more aggressive PSC subtype [38]. The key milestones in the development of savolitinib for NSCLC treatment are demonstrated in Figure 3.

## 3. First Steps towards the Development of Savolitinib as Mono and Combination Therapies

The availability of substantial evidence of anti-tumor activity and acceptable safety profile led to the development of savolitinib as a treatment for advanced NSCLC with *MET* aberrations. The high selectivity of savolitinib for *MET* was confirmed using a screening platform of more than 900 cell lines of which 111 represented NSCLC [39]. In vitro study by Henry and colleagues demonstrated the ability of savolitinib as a single agent to inhibit *MET* activity and reduce NSCLC cell viability in a dose dependent manner [39]. Further, anti-tumor efficacy was observed with savolitinib in vivo, in lung cancer PDX model with *MET*ex14 mutation. Savolitinib showed tumor regression (tumor volume reduction: 62%) with a dose of 25 mg/kg in all mice on treatment (*n* = 9) as well as 98% inhibition in tumor growth (TGI) with 5 mg/kg dose in 4 out of 9 mice of PDX model (Data on file). In addition, in vivo study using H1993 and EBC-1 tumor xenografts showed considerable decrease in tumor growth, with savolitinib achieving an optimal response at doses as low as 0.3 mg/kg and 2.5 mg/kg in H1993 and EBC-1 tumors, respectively [39]. Interestingly, the same group (Henry and colleagues) concurred that savolitinib resistance in NSCLC is partially driven by MYC overexpression in H1993 cells, suggesting potential mechanism and treatment strategies for future acquired resistance to MEK–TKI.

Savolitinib, in combination with erlotinib, a first-generation EGFR–TKI inhibitor, showed substantial tumor inhibition in H441, an *EGFR* wild type model with *MET* amplification [40,41]. In addition, savolitinib treatment exhibited substantial anti-tumor activity in vivo (tumor regression: 35%) in the NSCLC cancer cell line NCI-H820 harboring an activating *EGFR* mutation (Ex19del), a gefitinib/erlotinib resistant mutation (T790M) as well as hyperactivated *MET* (data on file). Osimertinib, a third-generation, irreversible EGFR–TKI, at either 25 mg/kg daily or 12.5 mg/kg daily exhibited minimal anti-tumor activity, with TGI of 24% and 4%, respectively. However, when treated in combination with savolitinib, 25 mg/kg of savolitinib plus osimertinib at either 25 mg/kg or 12.5 mg/kg daily resulted in 94% and 90% TGI, respectively. These preclinical results highlight the beneficial anti-tumor effect of osimertinib plus savolitinib combination at optimal doses of 0.3~1.5 mg/kg savolitinib combined with 10 mg/kg osimertinib. Further, another study analyzed different doses of savolitinib, ranging from 0.02 mg/kg to 15 mg/kg (15 mg/kg equivalent to 600 mg clinical dose), in combination with a fixed dose of 10 mg/kg osimertinib (equivalent to 80 mg clinical dose). Pan–CYP inhibitor 1–aminobenzotriazole was dosed along with savolitinib and osimertinib to prolong PK half-life by reducing elimination rate so that plasma concentration time profile matches clinical exposure of the drugs (data on file). The combination of osimertinib and savolitinib demonstrated strong anti-tumor activity leading to tumor regressions. The benefit of combination treatment was observed with as low as 0.3 mg/kg dose of savolitinib. Thus, these encouraging preclinical results led to the evaluation of savolitinib’s efficacy and safety in clinical trials for NSCLC with *MET* aberrations.

## 4. Clinical Development of Savolitinib: Phase I Trials

A first in-human phase I clinical study (NCT01773018) was conducted in patients (*n* = 48) with locally advanced solid tumors from Australia [42]. The doses administered were 100–1000 mg once daily (OD) and 300–500 mg twice-daily (BID), and the maximum tolerated dose was 800 mg. Savolitinib showed preliminary efficacy in patients with papillary renal cell carcinoma with *MET* gene copy number changes. The most frequent adverse events (AE) were nausea (62.5%), vomiting (41.7%), fatigue (35.4%) and peripheral edema (27.1%). The tolerability profile of savolitinib was acceptable, and the recommended phase II dose (RP2D) was established as 600 mg OD [42]. In another open-label, multi-center, phase Ia/Ib study (NCT0198555) conducted in China in patients (*n* = 85) with advanced tumors bearing *MET* aberrations, savolitinib demonstrated a manageable safety profile and promising anti-tumor activity in NSCLC with *MET*ex14 mutation, apparent tumor shrinkage (55% and 27%) in target lesions was observed, although partial response (PR) was not achieved. The most common treatment-related AEs were nausea (29.4%), vomiting (27.1%) and peripheral edema (21.2%). The RP2D of savolitinib was established at 600 mg OD or 500 mg BID and was consistent with phase I first-in human study conducted in Australia [43]. There was certain comparability between the patients with NSCLC enrolled in the phase I study conducted in Australia and the phase I study conducted in China, and thus the results could be analyzed accordingly [42,43].

Savolitinib demonstrated the ability to overcome *MET*-mediated resistance in patients with *EGFR*-mutant, *MET*-amplified or c-MET overexpressed NSCLC when combined with osimertinib, and these benefits extended to those with disease that had previously progressed on a prior EGFR–TKI [29,44]. Part A of the multi-arm phase Ib TATTON study (NCT02143466) demonstrated the safety and tolerability of osimertinib plus savolitinib (*n* = 18) in patients with advanced NSCLC disease progression on a prior EGFR–TKI [44]. Doses of savolitinib applied were escalated from 600 to 800 mg OD with a fixed dose of osimertinib 80 mg. The most common AEs reported were nausea (67%), rash (56%) and vomiting (50%). The objective response rate (ORR) was 44% [44]. Furthermore, in the expansion cohorts of TATTON trial, investigators evaluated the safety and efficacy of osimertinib plus savolitinib in locally advanced or metastatic, *MET*-amplified, *EGFR* mutation-positive NSCLC patients who had progressed on EGFR–TKIs [29]. Part B (*n* = 138) was substratified into three cohorts: B1 included those who had previously received a third-generation EGFR-TKI; patients without prior third-generation EGFR–TKI treatment were separated into B2 with Thr790Met negative and B3 with Thr790Met positive, at the time of enrolment. These patients received 600 mg QD, although the protocol was later amended, causing patients who weighed lesser than 55 kg to receive a 300-mg dose of savolitinib. The Part D expansion cohort was comprised of patients (*n* = 42) who had not previously received a third-generation EGFR–TKI and were T790M negative, and these patients received osimertinib 80 mg plus savolitinib 300 mg OD. Objective partial responses (PR) were observed (by 4 March 2020) in 68 (49%) patients in total of part B, with 23 (33%) patients, 33 (65%) patients and 12 (67%) patients in B1, B2 and B3, respectively, while in 26 (62%) patients in part D [45]. Regarding safety, the 4 expanded cohorts had similar safety profiles with 28% in part B and 19% in part D experiencing AEs possibly related to savolitinib. Serious AEs of grade 3 or 4 were associated with 49% patients in part B and 38% patients in part D. The most common AEs of grade 1–2 in expanded cohorts included nausea (48%), peripheral edema (34%), decreased appetite (32%), vomiting (30%) and fatigue (28%). In part B cohorts, the most common grade 3 or higher AEs related to savolitinib were decreased neutrophil count (6%) and aminotransferase elevations (4%), while in part D, hypersensitivity (5%), diarrhea (5%) and myalgia (5%) are more frequent [45]. Generally, in the dose expansion cohorts of TATTON trial, savolitinib plus osimertinib showed promising anti-tumor activity in *MET*-amplified *EGFR* positive advanced NSCLC patients who received a prior third-generation EGFR–TKI. These results have now been further investigated in the phase II SAVANNAH trial.

In another phase Ib study (NCT02374645), the clinical evaluation of savolitinib plus gefitinib (a first-generation EGFR-TKI) demonstrated promising anti-tumor activity with acceptable safety profile in *EGFRm*, *MET*-amplified advanced NSCLC patients from China who had disease progression on EGFR-TKIs. Patients received savolitinib 600 or 800 mg plus gefitinib 250 mg orally OD for which no dose-limiting toxicities were reported in safety run-in. The most commonly reported AEs were vomiting (46%), nausea (40%) and increased aspartate aminotransferase (39%) [30]. ORR in *EGFR* T790M-negative and -positive patients were 52% and 9%, respectively, suggesting beneficial anti-tumor activity [30].

## 5. Clinical Development of Savolitinib: Phase II Trials

A pivotal open-label phase II clinical study (NCT02897479) conducted in China demonstrated encouraging efficacy and tolerable safety profile of savolitinib in overall and patient subsets stratified according to tumor type (PSC and other NSCLC), brain metastasis status and prior anti-tumor treatment (pretreated and treatment naïve) [38]. Unresectable or metastatic NSCLC patients (*n* = 70) harboring *MET*ex14 mutation were administered savolitinib monotherapy at recommended starting dose of 600 mg orally once daily (OD) for patients weighing ≥50 kg, or 400 mg OD for patients weighing <50 kg, until disease progression or unacceptable toxicity. The majority of patients were elderly with advanced NSCLC on prior systemic therapy. In both the full analysis set (FAS) and the tumor response evaluable set (TRES), independent review committee (IRC) assessments were the main analyses, while investigators’ (INV) assessments were supportive analyses. The IRC-assessed tumor response evaluable set (TRES) was comprised of 62 patients. The ECOG performance status of full analysis set (FAS) for majority of patients (81%) was 1 and in pre-specified subsets (PSC vs. other NSCLC subtypes, treatment naïve vs. previously treated), 78% to 88% patients had ECOG status of 1. Of the total PSC population (*n* = 25), pre-treated and treatment-naïve subsets were comprised of 29% and 46%, while brain metastasis and non-brain metastasis groups were comprised of 13% and 42% of PSC patients, respectively [38,46,47]. The primary efficacy end point was ORR (as assessed by IRC in TRES) defined as the proportion of patients with a confirmed complete response or partial response according to RECIST version 1.1. Secondary outcomes included duration of response (DoR), time to response (TTR), progression free survival (PFS), overall survival (OS) and safety. The latest results of the trial were presented at the 2022 ELCC conference and published in JTO Clinical and Research Reports [46,48]. The baseline characteristics are provided in Table 2.

### 5.1. Efficacy Evidence

At a median follow-up of 17.6 months, the IRC and INV assessed ORR was 49.2% and 53.2 %, respectively in TRES subset, while ORR assessed in FAS set by IRC and INV was 42.9% and 47.1%, respectively. Further, the IRC and INV assessed disease control rate (DCR) was 93.4% and 91.9%, respectively in TRES subset, while DCR assessed in FAS set by IRC and INV was 82.9% and 81.4%, respectively. The median time to response was 1.4 months across TRES and FAS sets as judged by IRC and INV. Median DoR for TRES and FAS as assessed by IRC and INV was 8.3 and 6.9 months. Savolitinib was associated with mOS of 12.5 months and a mPFS of 6.9 months in FAS at a median follow-up time of 28.4 months. The 18-month OS rate is 42.1%, dropping to 31.5% at 24 months [38,48].

In subgroup analyses (assessed in TRES set by INV, median follow-up of 28.4 months), for PSC (*n* = 20), 10 patients had partial response (ORR 50%) with a median duration of response of 12.4 months. In other NSCLC subtypes (*n* = 42), 23 patients had partial response (ORR 54.8%) with a median duration of response of 5.6 months and DCR of 92.9%. In pre-treated (*n* = 38) patients, partial response was observed in 20 patients (52.6%), while in treatment-naïve (*n* = 24) subgroup, partial response was observed in 13 patients (54.2%). Extracranial ORR for brain metastasis group was 64.3%. For survival outcomes, the PSC group showed a mPFS of 5.5 months, while with brain metastasis (*n* = 15), it was 7.0 months and without brain metastasis was 6.2 months. Similar values of mPFS were observed with pre-treated (6.9 months) and treatment-naïve (6.9 months) subgroups, respectively. The mOS for PSC and other NSCLC patients was 10.6 months and 17.3 months, respectively, with corresponding 24-month OS rates of 26% and 35%. Among brain metastases patients, the mOS was 17.7 months with the 24-month OS rate being 36%. The mOS for pre-treated and treatment-naïve patients was 19.4 months and 10.9 months, respectively, with corresponding 24-month OS rates of 38% and 22% [48]. However, this large difference in OS can be attributed to the higher proportion of patients with PSC in treatment-naïve population (46% vs. 29% in pre-treated patients) and a higher median age (74.5 vs. 67.7 in pre-treated patients). Patients with PSC had a short mOS vs other NSCLC patient (10.6 months vs. 17.3 months), likely due to the poor prognosis associated with PSC. These results confirmed savolitinib having beneficial efficacy towards NSCLC with *MET*ex14 mutation and its PSC subtype [32,38,48]. The PFS and OS results have been illustrated graphically in Table 3.

An earlier study reported mOS of 6.7 months in patients with *MET*ex14 mutation NSCLC on chemotherapy treatment who did not receive prior targeted therapy [49]. In addition, mOS of PSC subset in NSCLC patients treated with chemotherapy has been reported to be 4 to 8 months [32,49,50,51,52,53]. With savolitinib, mOS of NSCLC patients reaches 12.5 months with 70% maturity. In PSC subset, higher OS is seen with savolitinib treatment compared to chemotherapy, with OS reaching 10.6 months. So far, literature related to MET inhibitor treatment with PSC population is available only for savolitinib [51,52,53,54].

P–glycoprotein (gp) and breast cancer resistance protein (BRCP) are efflux proteins located in the luminal membrane of brain capillary endothelium, preventing drugs from entering the central nervous system. Most MET inhibitors, such as crizotinib and tepotinib, are known substrates of the P–gp and BRCP efflux transport system [55,56,57]. Steady concentrations of savolitnib are readily maintained in an intracerebral area which may be attributed to it not being a substrate of P–gp and BRCP efflux transport system. Promising efficacy of savolitinib was observed in brain metastasis subgroup, with ORR at 64.3%, DCR at 100% and significant survival benefit (PFS, 7.0 months; OS, 17.7 months). These encouraging results provide a treatment option for this subgroup of patients with poor prognosis and few treatment options [38,47,48].

### 5.2. Safety Evidence

Savolitinib demonstrated tolerable safety profile consistent with previous trials; most AEs were grades 1–2 and resolved with dose adjustment and discontinuation. Adverse events that presented at rates of ≥30% are listed below (Table 4, median follow-up of 28.4 months). The incidence of grade3 or more AEs was 65.7%, while 50% of patients reported treatment-related serious adverse events (SAE). The top ≥ grade 3 AE was elevated AST (12.9%). The most common treatment-related AEs (TRAEs) (≥30%) are peripheral edema (55.7%), nausea (45.7%) and elevated aminotransferase (38.6% and 37.1%). The top ≥ grade 3 treatment related AE was elevated AST (12.9%) [48,58]. The common SAEs reported were abnormal liver function (4.3%, 3 patients), drug hypersensitivity reaction (2.9%, 2 patients) and fever (2.9%, 2 patients). Treatment related fatal SAE, tumor lysis syndrome was reported in one patient. Ten patients discontinued treatment due to AEs, of which drug-induced liver damage and drug hypersensitivity reactions were seen in 2.9% of patients (2 patients), respectively [38]. No occurrence of pulmonary interstitial pneumonia and interstitial lung disease (ILD) was observed with savolitinib, while ILD is seen with tepotinib (*n* = 2) and capmatinib (*n* = 1) [4,59].

The updated results further confirm that savolitinib can benefit *MET*ex14-mutated NSCLC patients and each subgroup with acceptable safety profile [38,46,47,48]. Savolitinib thus displays promising efficacy and tolerability in PSC associated with *MET*ex14 mutation and holds potential to become the first approved treatment in this setting. In addition, the study showed that savolitinib can penetrate the blood–brain barrier and is effective in patients with brain metastases.

### 5.3. Brief Introduction of Other Phase II Trials

Other ongoing phase II trials include SAVANNAH, SOUND and FLOWERS trials. SAVANNAH trial (NCT03778229) continues to explore the sequence of savolitinib plus osimertinib with previous osimertinib monotherapy resistance. It is a phase II, single-arm study evaluating the efficacy of osimertinib in combination with savolitinib in 259 patients with *EGFRm* and *MET* amplified and/or c-MET overexpressed locally advanced or metastatic NSCLC who have progressed on osimertinib. Patients were treated with osimertinib (80 mg OD) and savolitinib (300 mg QD, 300 mg BID or 600 mg OD) until objective disease progression. Efficacy endpoints—such as ORR (primary endpoint), PFS, OS, DoR, HRQoL, pharmacokinetics, safety points such as AEs and patient related outcomes (PROs)—were studied. This is the first phase II clinical study of the third-generation EGFR–TKI osimertinib resistance in patients with advanced NSCLC with *MET* amplification and/or c-MET overexpression. *MET* detection was performed using fluorescence in situ hybridization (FISH) and immunohistochemistry (IHC) methods. The detection criteria were set to FISH, *MET* GCN ≥ 5 and/or *MET/*CEP7 ≥ 2; IHC, ≥50% tumor cells 3+. Sixty-two percent of osimertinib resistant patients was at low threshold [IHC50+ and/or FISH5+] as well as 34%—at the high threshold [IHC90+ and/or FISH10+] subgroups. Figure S1 provides the proportion of patient population with *MET* amplified and/or c-MET overexpressed in this study suggesting amplification and/or overexpression is the most common osimertinib resistance mechanism. The baseline characteristics are provided in Table S1. The overall median age of patients is 63 years, 62% were female, 54% were Asian and 34% were with brain metastases at baseline. On savolitinib 300 mg OD plus osimertinib 80 mg OD treatment, advanced NSCLC patients (*n* = 193) with high *MET* amplification and/or high threshold c-MET overexpression level show a trend toward better efficacy benefit, emphasizing the necessity of patients’ selection according to appropriate *MET* detection criteria in this population. Among the overall population, ORR was 32%; median DoR was 8.3 months; and median PFS was 5.3 months, while among 108 patients who met the threshold for high *MET* amplification and/or high threshold c-MET overexpression level (IHC90+ and/or FISH10+), ORR was 49%; median DoR was 9.3 months; and median PFS was 7.1 months (Table S2). The safety results showed that the incidence of treatment-related AEs was 84%; treatment-related ≥grade 3 AEs at 20%; and treatment-related SAEs at 7% (Table S3). The incidence of hypersensitivity, ILD and pneumonia were 2% (4/196), and QT interval prolongation at 5% (10/196) [60].

In addition, the FLOWERS trial (NCT05163249) explores the efficacy and safety of osimertinib with or without savolitinib in patients with de novo *MET* amplified and/or c-MET overexpressed, *EGFR*-mutant advanced NSCLC. In SOUND trial (NCT05374603), an open-label, interventional, multi-center, exploratory trial, savolitinib combined with durvalumab will be evaluated in Chinese *EGFR* wild-type locally advanced or metastatic NSCLC patients with *MET* alterations. NSCLC patients from China with *MET* amplification (*n* = 30) and *MET*ex14 mutation (*n* = 30) will be treated with 1500 mg durvalumab and 300 to 600 mg savolitinib (OD) for 28-day/cycle till disease progression, death or toxicity. Efficacy endpoints will be PFS, ORR, DoR, DCR, 12 m OS rate and safety endpoints will be AEs and AEs of special interest (AESI) [61]. Further, phase III SAFFRON trial (NCT05261399) is investigating savolitinib plus osimertinib versus platinum-based doublet chemotherapy in participants with NSCLC (*EGFR* mutated, c-MET overexpressed and/or *MET* gene amplified) who have progressed on osimertinib treatment.

## 6. Ongoing Phase III Trials

Currently, four phase III trials evaluating savolitinib as a monotherapy and in combination with EGFR-TKIs are underway. The confirmatory phase IIIb clinical study (CTR20211151) is evaluating efficacy and safety of savolitinib in two cohorts from patients with locally advanced or metastatic NSCLC with *MET*ex14 mutation in China; patients of one cohort are with disease progression or toxicity intolerance after previous platinum-based chemotherapy regimens, and patients of another cohort are with no prior systemic antineoplastic therapy for advanced disease. The patients were treated until disease progression or intolerable toxicity. Phase III SACHI trial (CTR20211441) is a randomized, two-arm, open-label, multi-center study evaluating the efficacy and safety of savolitinib plus osimertinib versus chemotherapy in NSCLC patients from China with *MET* amplification who has progressed after first- to third-generation EGFR–TKI therapy and has already begun its recruitment in multiple centers. Another similar phase III trial SAFFRON is designed to evaluate the efficacy and safety of the same combined therapy as SACHI versus chemotherapy, but focus on global advanced NSCLC patients with *MET* amplification/c-MET overexpression that progressed after osimertinib treatment. SANOVO Phase III study is evaluating the efficacy and safety of savolitinib in combination with osimertinib in treatment-naïve patients with *EGFR* mutant positive and c-MET overexpression advanced NSCLC (NCT05009836).

## 7. Discussion

Savolitinib, an investigational MET highly selective agent, has shown pronounced efficacy in preclinical and clinical studies. Savolitinib demonstrated preclinical anti-tumor activity against *MET*-dependent cancer cell line growth and *MET*-driven tumor growth in xenograft models. Following which, data from a phase I clinical trial established recommended phase II dose in patients with *MET*ex14-mutated NSCLC. Further, the TATTON study established utility of savolitinib with osimertinib in advanced NSCLC with *MET*-mediated acquired resistance to EGFR-TKIs. Final results of the phase II study (NCT02897479) further confirmed the benefit of savolitinib in patients with *MET*ex14-mutated NSCLC across all predefined subgroups. In addition, phase IIIb clinical study CTR20211151 is confirming the result of phase II study on *MET*ex14-mutated NSCLC, while three ongoing phase II trials, SAVANNAH, SOUND and FLOWERS, as well as three phase IIIB trials, SAFFRON, SACHI and SANOVO, are actively exploring solutions for different types of savolitinib combination regimens against EGFR resistance mechanisms. Preliminary results of the SAVANNAH trial have demonstrated the beneficial efficacy of osimertinib plus savolitinib in *EGFRm* NSCLC patients with *MET* amplified and/or c-MET overexpressed, supporting the results of TATTON study and paving the way for phase III SACHI and SAFFRON study.

In the hallmark phase II registry trial, savolitinib displayed promising efficacy and tolerability in patients with *MET*ex14-altered advanced NSCLC, with mOS reaching 12.5 months. The effect of savolitinib was rapid, substantiated by time to response (TTR) of 1.4 months. Promising results with PFS of 5.5 months and OS of 10.6 months were also seen in the PSC subtype, which does not respond well to chemotherapy and has limited effective treatments. By now, savolitinib is the only MET inhibitor with data related to PSC associated with *MET*ex14 mutation and is becoming the first approved agent in this setting. For patients of treatment naïve population, the PFS and OS of savolitinib were 6.9 months and 10.9 months, respectively, while PFS and OS of prior treatment patients reached 6.9 months and 19.4 months, respectively. In the current scenario, the reported ORR of savolitinib is the highest in the prior treatment population compared to other treatments (52.6% vs. 44.0% of capmatinib, 49.5% of tepotinib and 21% of amivantamab) [48,62,63,64]. Savolitinib is also currently the only MET inhibitor that has recorded beneficial OS data in brain metastases, with PFS of 7.0 months and OS of 17.7 months. In addition, savolitinib has the best tumor response in brain metastasis population with ORR at 64.3% and DCR at 100% [38,48]. Based on these promising results, savolitinib received its first conditional approval by NMPA, China in June 2021, for patients with *MET*ex14-altered NSCLC after systemic treatment resistance or unable to receive chemotherapy. Post-marketing phase IIIb trial is now undergoing (HutchMed) in larger population of NSCLC patients and is expected to provide more clinical evidences for savolitinib in first-line therapy. Furthermore, latest *post hoc* analysis based on ctDNA detection suggests undetectable baseline *MET*ex14 or post-treatment clearance in ctDNA being relevant to favourable clinical outcomes, including better PFS and OS results, while secondary *MET* mutations and other acquired gene alterations after treatment (e.g., RTK–RASP–I3K pathway) may explain resistance mechanism to savolitinib [65].

Table 5 summarizes the data for MET-TKIs developed for *MET*ex14-altered advanced NSCLC population as well as subtypes [4,38,47,48,58,59,62,63,64,66,67,68,69,70,71,72,73]. Patient population of Chinese Phase II registry trial were from China. In other global trials, east Asian population varied from 15.9 to 50.9%. Proportion of NSCLC patients with brain metastases was higher (28.9%) in Chinese Phase II registry trial compared to other trials [48]. Tumor response of different types of MET–TKIs shows ORR (54.8%) and DCR (92.9%) to be highest with savolitinib. Among AEs, most commonly, elevated transaminases were seen with savolitinib, tepotinib and crizotinib; peripheral edema with savolitnib, capmatinib and tepotinib; ILD with capmitnib, tepotinib and crizotinib; difficulty in breathing in tepotinib, crizotinib and amivantamab [38,48,58,59,62,63,64,67,68,69,70,71,72,73].

Bypass activation mediated by the *MET* signaling pathway is one of the important mechanisms leading to EGFR–TKI resistance. *MET*-driven resistance can be manifested as gene-level amplification or protein-level overexpression with previous treatment regimens such as chemotherapy, immunotherapy and targeted therapies including *EGFR*, *BRAF* and *MEK* [74,75]. The efficacy of tepotinib on NSCLC with T790M-negative *MET* amplification and/or c-MET overexpression after first/second-generation EGFR–TKI resistance is limited, with a mPFS of only 4.9 months [76,77]. The current immunotherapy efficacy for advanced NSCLC after EGFR–TKI resistance needs further improvement, and there is a lack of *MET* amplification and/or c-MET overexpression subgroup data. Nivolumab monotherapy has limited efficacy after EGFR–TKI resistance, with a mPFS of only 1.5–1.7 months [78,79]. In IMpower 150 and ORIENT-31 studies, EGFR–TKI resistance, followed by immunotherapy combined with bevacizumab and chemotherapy, showed a mPFS of 6.9–9.7 months but no subgroup data on *MET* amplification and/or c-MET overexpression was reported; meanwhile, safety of the combination therapy regimen needs attention [80,81]. *MET*-amplified and/or c-MET overexpressed advanced NSCLC patients with EGFR–TKI resistance have limited therapeutic effect with MET inhibitor monotherapy. Only 1 of 12 evaluable patients on inhibitor monotherapy reported an objective response [82]. Dual-target inhibition of *EGFR* and *MET* pathways may bring synergistic therapeutic benefit in *MET*-driven EGFR–TKI-resistant advanced NSCLC patients [83]. Meanwhile, efficacy of savolitinib combined with durvalumab in *EGFR* wild-type NSCLC with *MET* alterations is also under exploration in SOUND trial, as previously described.

The combination of EGFR inhibitor and MET-highly selective TKI possesses the potential to prevent or overcome *MET*-driven resistance to EGFR–TKIs. Acquired resistance to first- and second-generation EGFR–TKIs is often caused by the acquisition of the T790M mutation, which accounts for approximately 60% of resistant cases and has been overcome by third-generation EGFR–TKIs such as osimertinib. For first- and second-generation EGFR–TKIs, acquired resistance for *MET*-amplification is at least 5% (for example, gefitinib), while up to 25% of acquired resistance is observed with third generation EGFR–TKI (for example, osimertinib) [84]. TATTON study, set up in the back drop of acquired *MET* amplification associated with EGFR–TKI resistance offered explicit benefit with savolitinib in NSCLC patients without prior third-generation EGFR–TKI, while those who were administered with a prior third-generation EGFR–TKI had a relatively lower rate of response regardless of T790 status, possibly related to larger proportion of patients with ≥3 lines of treatment comprising the prior third-generation EGFR–TKI group (56.5% vs. 22.6% in partB2 + partD). Nonetheless, TATTON program demonstrated beneficial efficacy of savolitinib plus osimertinib combination in the *MET*-amplified, *EGFR* mutation–positive setting with acceptable safety profile which is a first in this setting [44]. Further, SAVANNAH phase II trial validates TATTON results with advanced NSCLC patients with *MET* amplification or c-MET overexpression due to osimertinib-acquired resistance. Initial results from the SAVANNAH trial show a trend toward improved response rates, with increasing level of *MET* amplified and/or c-MET overexpressed. Across all patients in this analysis, ORR was 32%; mDoR was 8.3 months; and mPFS was 5.3 months, while in high level *MET* amplification and/or c-MET overexpression subgroup, ORR was 49%; mDoR was 9.3 months; and mPFS was 7.1 months [60]. A summary of key data after EGFR–TKI resistance with secondary *MET* alterations treated with combination therapies available so far is provided in Table 6.

*MET* amplification can be detected by using fluorescence in situ hybridization (FISH) and immunohistochemistry (IHC). With *MET* amplification, *MET/CEP7* ratio is as follows: low: ≥1.8 to ≤2.2; intermediate: >2.2 to <5; or high: ≥5 will be applied in clinical settings when treating patients with MET inhibitors [90]. The frequency of *MET* amplification in NSCLC ranges from 3% to 10% depending on the cut-off of *MET* copies per cell [91]. c-MET overexpression score of 2+ or 3+ as determined by IHC is considered as *MET* positive [60]. The TATTON study conducted an exploratory analysis of the relationship between the *MET* detection method and the dual-target efficacy after third-generation EGFR–TKI resistance: Based on FISH detection, the ORR value of *MET* local amplification was higher than that of *MET* polysomy patients although polysomy patients benefited from the treatment. In the *MET*-amplified population, patients with higher gene copy numbers detected by FISH had a better treatment benefit [44]. Further in SAVANNAH trial, promising clinical efficacy in a population with high *MET* amplification and/or high threshold c-MET overexpression level (IHC 90+ and/or FISH 10+) with an ORR 49%, mDoR of 9.3 months, and mPFS of 7.1 months was observed. The safety profile was acceptable, similar to that of TATTON study [60]. Further results of the SAVANNAH trial are awaited. However, the sample sizes of these studies are limited. Hence the need to interpret the results with caution is warranted, and further verification is required with larger clinical trials. Further phase III confirmatory trials, SAFFRON and SACHI have been initiated in patients whose disease progressed following treatment with any EGFR-TKI.

## 8. Conclusions

The conditional approval of savolitinib for the treatment of metastatic *MET*ex14-mutated NSCLC is based on encouraging results from phase 2 trial conducted in China including patients with the more aggressive PSC subtype and brain metastasis. Savolitinib is a potent, highly selective MET inhibitor with robust response in advanced NSCLC. Preclinical and clinical data have shown savolitinib as effective and tolerable treatment in advanced NSCLC patients with *MET*ex14 skipping mutations. When used in combination with EGFR-TKIs, savolitinib has the potential to overcome resistance to these treatments driven through *MET* amplifications and/or c-MET overexpression, with future clinical trials verification needed. In conclusion, savolitinib offer another promising targeted treatment in the paradigm of metastatic NSCLC.

## Figures and Tables

**Table 1:** Proportion of different *MET* alterations in NSCLC patients.

*MET* Alterations		Proportion, %	Publication [Reference]
***MET*ex14**	NSCLC ^1^	0.9–4	Davies KD et al. [6]
	PSC subtype	20–31.8	Mo HN et al. [15] Tong JH et al. [16]
*MET* Fusion ^1^		0.5	Recondo G et al. [18]
MET Overexpression ^1^		13.7–63.7	Guo R et al. [17]
*MET* Amplification ^2^		1–5	Guo R et al. [17]
Secondary *MET* Amplification	1/2G EGFR–TKI resistance	5–22	Recondo G et al. [18] Bean J et al. [21]
	3G EGFR–TKI resistance	5–50	Wang Y et al. [22]
*MET* Amplification Co-occurrence with *EGFR* Mutation ^3^		2–11	Li XM et al. [23] Lai GGY et al. [24]

^1^ Based on overall NSCLC population. ^2^ Based on treatment-naïve NSCLC population. ^3^ Based on *EGFR*-positive treatment-naïve NSCLC population. EGFR, Epidermal Growth Factor Receptor; TKI, Tyrosine Kinase Inhibitor; MET, Mesenchymal Epithelial Transition; NSCLC, Non-Small Cell Lung Cancer; PSC, Pulmonary Sarcomatoid Carcinoma.

**Table 2:** Baseline characteristics of phase II trial conducted in China [48].

		Age			Sex		Smoking History		ECOG Performance Status		
		Median Age, Years	<75 Years	≥75 Years	Female	Male	Non-Smokers	Smokers	0	1	3
**Full Analysis Set** **(*n* = 70)**		68.7 (51.7–85.0)	54 (77%)	16 (23%)	29 (41%)	41 (59%)	42 (60%)	28 (40%)	12 (17%)	57 (81%)	1 (1%)
Type of Primary Tumor	PSC (*n* = 25)	69.3 (54.1–84.8)	19 (76%)	6 (24%)	8 (32%)	17 (68%)	13 (52%)	12 (48%)	3 (12%)	22 (88%)	0
	Other NSCLC (*n* = 45)	68.1 (51.7–85.0)	35 (78%)	10 (22%)	21 (47%)	24 (53%)	29 (64%)	16 (36%)	9 (20%)	35 (78%)	1 (2%)
Prior Anti-tumor Treatment	Pre-treated (*n* = 42)	67.7 (51.7–84.8)	38 (90%)	4 (10%)	17 (40%)	25 (60%)	28 (67%)	14 (33%)	8 (19%)	34 (81%)	0
	Treatment-naïve (*n* = 28)	74.5 (56.0–85.0)	16 (57%)	12 (43%)	17 (40%)	16 (57%)	14 (50%)	14 (50%)	4 (14%)	23 (82%)	1 (4%)
Brain Metastases Status	Brain metastases (*n* = 15)	68.6 (51.7–84.8)	11 (73%)	4 (27%)	7 (47%)	8 (53%)	11 (73%)	4 (27%)	3 (20%)	12 (80%)	0
	Non-brain metastases (*n* = 55)	68.7 (51.9–85.0)	43 (78%)	12 (22%)	22 (40%)	33 (60%)	31 (56%)	24 (44%)	9 (16%)	45 (82%)	1 (2%)
		**Histology**						**Prior Anti-tumor Treatment**		**Brain Involvement at Baseline**	
		Pulmonary sarcomatoid carcinoma	Other NSCLC subtypes					Yes	No		
			Adenocarcinoma	Squamous cell carcinoma	Adenosquamous carcinoma		NSCLC, not otherwise specified				
Full Analysis Set (*n* = 70)		25 (36%)	40 (57%)	3 (3%)	1 (1%)		1 (1%)	42 (60%)	28 (40%)	15 (21%)	
Type of Primary Tumor	PSC (*n* = 25)	25 (100%)	-					12 (48%)	13 (52%)	2 (8%)	
	Other NSCLC (*n* = 45)	-	40 (89%)	3 (7%)	1 (2%)		1 (2%)	30 (67%)	15 (33%)	13 (29%)	
Prior Anti-tumor Treatment	Pre-treated (*n* = 42)	12 (29%)	27 (64%)	2 (5%)	1 (2%)		0	42 (100%)	-	11 (26%)	
	Treatment-naïve (*n* = 28)	13 (46%)	13 (46%)	1 (4%)	0		1 (4%)	-	28 (100%)	4 (14%)	
Brain Metastases Status	Brain metastases (*n* = 15)	2 (13%)	13 (87%)	0	0		0	11 (73%)	4 (27%)	15 (100%)	
	Non-brain metastases (*n* = 55)	23 (42%)	27 (49%)	3 (5%)	1 (2%)		1 (2%)	33 (60%)	22 (40%)	-	

Data in median (IQR) or *n* (%). NSCLC, Non-Small Cell Lung Cancer; PSC, Pulmonary Sarcomatoid Carcinoma; ECOG, Eastern Cooperative Oncology Group.

**Table 3:** Investigator-Assessed Responses in the Tumor-Response-Evaluable Set and the Full Analysis Set of Phase II Trial Conducted in China [48].

		ORR, *n* (%)	DCR, *n* (%)	Median DOR, Months ^1^	Median TTR, Months ^1^
Tumor-Response-Evaluable Set (*n* = 62)	Total (*n* = 62)	33 (53.2%)	57 (91.9%)	6.9	1.4
	PSC (*n* = 20)	10 (50.0%)	18 (90.0%)	12.4	1.4
	Other NSCLC subtypes (*n* = 42)	23 (54.8%)	39 (92.9%)	5.6	1.4
	Pretreated (*n* = 38)	20 (52.6%)	34 (89.5%)	10.9	1.4
	Treatment-naive (*n* = 24)	13 (54.2%)	23 (95.8%)	5.6	1.4
	Brain metastases (*n* = 14)	9 (64.3%)	14 (100.0%)	4.9	1.5
	Non-brain metastases (*n* = 48)	24 (50.0%)	43 (89.6%)	7.0	1.4
Full Analysis Set (*n* = 70)	Total (*n* = 70)	33 (47.1%)	57 (81.4%)	*n*/A	*n*/A
	PSC (*n* = 25)	10 (40.0%)	18 (72.0%)	*n*/A	*n*/A
	Other NSCLC subtypes (*n* = 45)	23 (51.1%)	39 (86.7%)	*n*/A	*n*/A
	Pretreated (*n* = 42)	20 (47.6%)	34 (81.0%)	*n*/A	*n*/A
	Treatment-naive (*n* = 28)	13 (46.4%)	23 (82.1%)	*n*/A	*n*/A
	Brain metastases (*n* = 15)	9 (60.0%)	14 (93.3%)	*n*/A	*n*/A
	Non-brain metastases (*n* = 55)	24 (43.6%)	43 (78.2%)	*n*/A	*n*/A

^1^ DOR and TTR were analyzed in the tumor-response-evaluable set. DCR, disease control rate; DOR, duration of response; *n*/A, not applicable; NSCLC, non-small cell lung cancer; ORR, objective response rate; PSC, pulmonary sarcomatoid carcinoma; TTR, time to response.

**Table 4:** Adverse events (>30%) in the full analysis set of phase II trials conducted in China (*n* = 70) [48].

		Any Grade	≥Grade 3
All-cause adverse events	Any event	70 (100.0%)	46 (65.7%)
	Peripheral edema	40 (57.1%)	6 (8.6%)
	Nausea	37 (52.9%)	0
	Hypoalbuminemia	29 (41.4%)	1 (1.4%)
	Elevated alanine aminotransferase	27 (38.6%)	7 (10.0%)
	Elevated aspartate aminotransferase	27 (38.6%)	9 (12.9%)
	Decreased appetite	24 (34.3%)	0
	Vomiting	23 (32.9%)	0
	Pyrexia	21 (30.0%)	1 (1.4%)
Treatment-related adverse events	Any event	70 (100.0%)	32 (45.7%)
	Peripheral edema	39 (55.7)	6 (8.6)
	Nausea	32 (45.7)	0
	Hypoalbuminemia	16 (22.9)	0
	Elevated alanine aminotransferase	27 (38.6)	7 (10.0%)
	Elevated aspartate aminotransferase	26 (37.1)	9 (12.9%)
	Decreased appetite	14 (20.0%)	0
	Vomiting	18 (25.7%)	0
	Pyrexia	11 (15.7%)	1 (1.4%)

Data in *n* (%). Derived from latest safety analysis of phase II trial (NCT02897479) [48].

**Table 5:** Data summary of MET inhibitors in *MET*ex14 mutation.

	Savolitinib ^1^	Capmatinib ^2^	Tepotinib ^3^	Crizotinib ^4^	Amivantamab ^5^
Approval	China approved in June 2021	Approved in the US in 2020	Approved in Japan in 2020	FDA breakthrough therapy designation	Approved in the US in May 2021
Mechanism	METi Ib	METi Ib	METi Ib	ALK/ROS1/METi Ia	Anti-MET and EGFR antibody
*n*	45	160	313	25/69	46
Population	100% Chinese patients	20.2% Asian patients	33.9% Asian patients	Unknown/15.9% Asian patients	50.9% Asian patients
Proportion of brain metastases	28.9%	16.9%	18.2%	Unknown	18.2%
Dose	600 mg (BW ≥ 50 kg), or 400 mg (BW < 50 kg) OD	400 mg BID	500 mg OD	250 mg BID	1050 mg (<80 kg), or 1400 mg (≥80 kg)
ORR	54.8%	52.5%	50.8%	12.0%/32.3%	32.6%
DCR	92.9%	88.1%	75.4%	44.0%/unknown	76.1%
Median PFS, Months	6.9	12.4/12.5/5.4/6.9	11.2	3.6/7.3	6.7
Common Grade 3/4 AEs	Elevated AST Elevated ALT Peripheral edema (No interstitial lung disease occurred in registry studies)	Peripheral edema Difficulty breathing Fatigue Elevated ALT Weak Pneumonia	Peripheral edema Generalized edema Vomit Nausea Interstitial lung disease	Elevated transaminases Difficulty breathing Hypophosphatemia Lymphopenia Pulmonary embolism Interstitial lung disease	Rash Hypoalbuminemia Difficulty breathing

^1^ The number of patients and the proportion of patients with brain metastases are based on other types of NSCLC in general, and the ORR, DCR and median PFS data are derived from data from other types of NSCLC in the efficacy-evaluable set [48]; safety data is analyzed based on the overall patient (*n* = 70) [38,48]. ^2^ Data derived from the latest analysis of four different cohorts from GEOMETRY mono-1 study: cohort 4, expansion cohort 6, cohort 5b and expansion cohort 7. Number of patients, proportion of brain metastases, ORR and DCR represent four cohorts in total; proportion of population based on cohort 4, 5b and 7; mPFS reflect results of four cohorts, respectively [62,71]. ^3^ Data based on VISION study cohort A + cohort C latest overall analysis [63]. ^4^ Patient population, number of patients, ORR, DCR and median PFS data are derived from two different studies of AcSé [72] and PROFILE-1001 [73]; safety data is based on combination of these two trials. ^5^ Data from latest analysis of CHRYSALIS study [64]. EGFR, Epidermal Growth Factor Receptor; TKI, Tyrosine Kinase Inhibitor; MET, Mesenchymal Epithelial Transition; ALK, Anaplastic Lymphoma Kinase; ROS1, ROS proto-oncogene 1; OD, Once Daily; BID, twice daily; ORR, Objective Response Rate; DCR, Disease Control Rate; PFS, Progression Free Survival; AE, Adverse Event; AST, Aspartate aminotransferase; ALT, Alanine aminotransferase.

**Table 6:** Summary of key data after EGFR–TKI resistance with secondary *MET* alterations treated with combination therapies.

Combination	Publication [Reference]	*n*	Patient Population	*MET* Status	ORR	Median PFS, Months
-	Sequist LV et al. [29] Hartmaier RJ et al. [45] ^1^	93	1/2G EGFR–TKI resistance, T790M-	FISH: *MET* GCN ≥ 5 or *MET/*CEP7 ≥ 2; IHC: 3+ in ≥50% tumor cell; NGS: ≥ 20% tumor cell, ≥200X seq, GCN ≥ 5	Part B2: 64.7%	Part B2: 9.1
					Part D: 61.9%	Part D: 9.0
	Hartmaier RJ et al. [45] ^2^	69	3G EGFR–TKI (osimertinib) resistance	FISH: *MET* GCN ≥ 5 or *MET/*CEP7 ≥ 2; IHC: 3+ in ≥50% tumor cell; NGS: ≥ 20% tumor cell, ≥200X seq, GCN ≥ 5	33.3%	5.5
	Yu HA et al. [85]	17	3G EGFR–TKI (osimertinib) resistance	NGS: GCN range from 7 to 68	41.2%	Unknown
	Ahn MJ et al. [60]	193	3G EGFR–TKI (osimertinib) resistance	FISH: *MET* GCN ≥ 5 or *MET/*CEP7 ≥ 2; IHC: 3+ in ≥50% tumor cell	Overall: 32%	Overall: 5.3
					FISH10+ or IHC90+: 49.1% ^3^	FISH10+ or IHC90+: 7.1
Capmatinib + Gefitinib	Wu YL et al. [86]	100	1/2G EGFR–TKI resistance, T790M-	FISH: GCN ≥ 4	4 ≤ GCN < 6: 22.2%	4 ≤ GCN < 6: 5.4
					GCN ≥ 6: 47.2%	GCN ≥ 6: 5.5
				IHC: 3+ in ≥50% tumor cell	IHC3+: 32.1%	IHC3+: 5.5
Tepotinib + Gefitinib	Wu YL et al. [87] Liam CK et al. [88]	31	1/2G EGFR–TKI resistance, T790M-	FISH: GCN ≥ 5 or *MET/*CEP7 ≥ 2	Overall: 45.2%	Overall: 4.9
					*MET* amp: 66.7%	*MET* amp: 16.6
				IHC: 2+ or 3+	IHC3+: 68.4%	IHC3+: 8.3
Amivantamab + Lazertinib	Bauml J et al. [76]	45	3G EGFR–TKI (osimertinib) resistance, without previous chemotherapy	No *MET* selection	Overall: 35.6%	Overall: 4.9
					*EGFR/MET* dependent: 47.1% ^4^	*EGFR/MET* dependent: 6.7
					Unknown/non-*EGFR/MET*: 28.6%	Unknown/non-*EGFR/MET*: 4.1
					EGFR/MET IHC+: 90.0%	EGFR/MET IHC+: 12.5
Telisotuzumab vedotin + Osimertinib	Goldman JW et al. [89]	19	3G EGFR–TKI (osimertinib) resistance	IHC: 3+ in ≥25% tumor cell	57.9%	Unknown

^1^ Data based on part B2 (*n* = 51) and part D (*n* = 42) of TATTON study. ^2^ Data based on part B1 of TATTON study. ^3^ Represents high *MET* amplification and/or high c-MET overexpression subgroup (*n* = 108); FISH10+: *MET* GCN ≥ 10; IHC90+: 3+ in ≥90% tumor cell. ^4^ *EGFR/MET* dependent, *EGFR/MET* dependent mechanism of resistance (*n* = 17); unknown/non-*EGFR/MET*, unknown mechanism or non-*EGFR/MET* mechanism of resistance to osimertinib (*n* = 28); EGFR/MET IHC+, high IHC results (combined EGFR + MET H score > 400) (*n* = 10). EGFR, Epidermal Growth Factor Receptor; TKI, Tyrosine Kinase Inhibitor; MET, Mesenchymal Epithelial Transition; FISH, Fluorescence In Situ Hybridization; IHC, Immunohistochemistry; GCN, Gene Copy Number; CEP7, Centromere 7; ORR, Objective Response Rate; PFS, Progression Free Survival.

## Supplementary Materials

The following supporting information can be downloaded at: https://www.mdpi.com/article/10.3390/cancers14246122/s1, Figure S1: Proportion of *MET*-altered NSCLC patients using different detection methods in SAVANNAH trail; Table S1: Baseline characteristics and clinical demographics of SAVANNAH study; Table S2: Efficacy parameters of SAVANNAH study; Table S3: Safety results of SAVANNAH trial.
