# Supplementary material for: Landscape of Savolitinib Development for the Treatment of Non-Small Cell Lung Cancer with MET Alteration—A Narrative Review
Source: Cancers (Basel). 2022 Dec 12;14(24):6122. doi: 10.3390/cancers14246122 (PMC9776447; doi:10.3390/cancers14246122)
Supplement: Supplementary file 1 [file cancers-14-06122-s001.zip › cancers-2052355-supplementary.pdf]

Supplementary Materials

Figures:

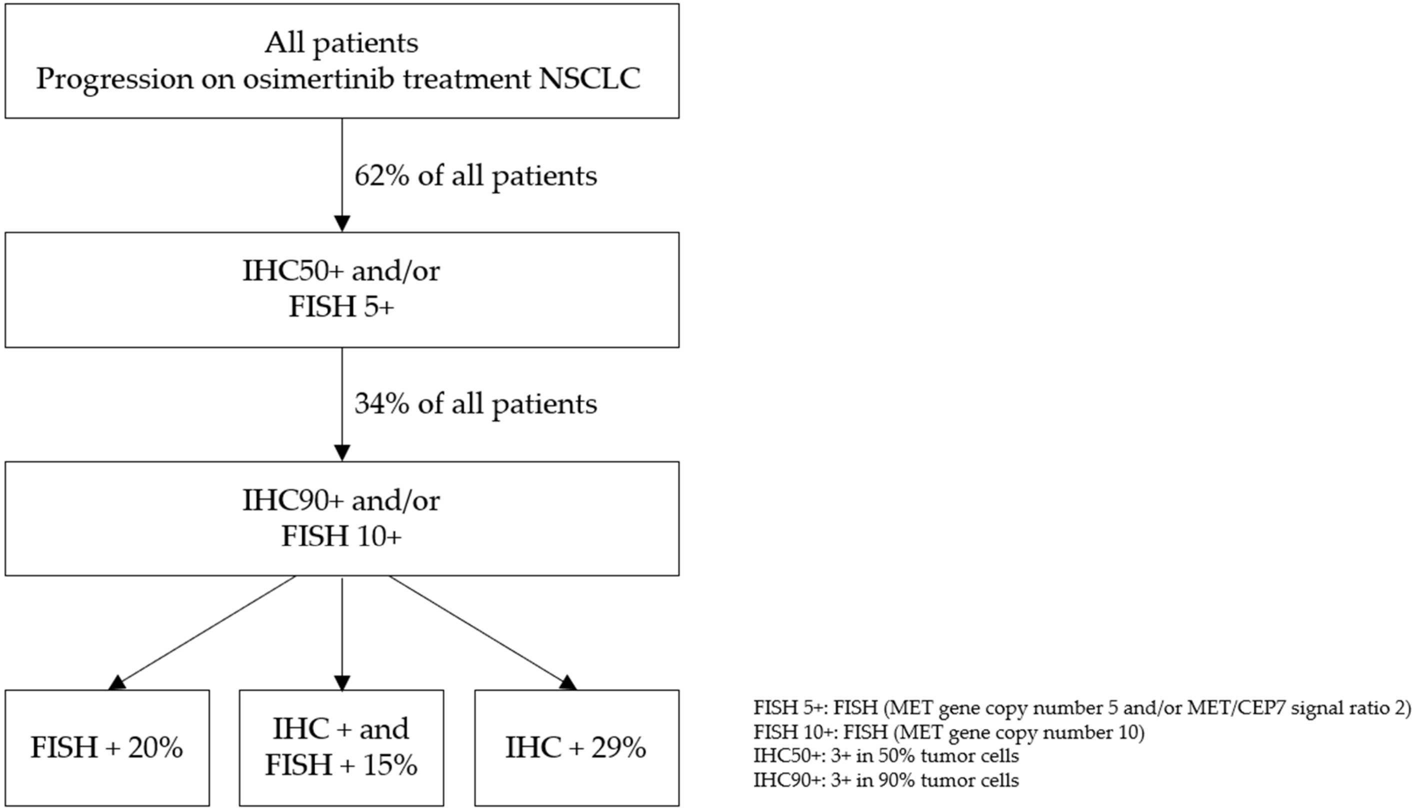

Figure S1: Proportion of *MET*-altered NSCLC patients using different detection methods in SAVANNAH trail [65].

Tables:

Table S1: Baseline characteristics and clinical demographics of SAVANNAH study [65].

Parameters	All patients; IHC50+ and/or FISH5+ (n=193*)		IHC90+ and/or FISH10+ Subgroup (n=109)		IHC90+ and/or FISH10+ (n=79)	
	**All (n=196)**	**No history of chemotherapy (n=160)**	**All (n=109)**	**No history of chemotherapy (n=88)**	**All (n=79)**	**No history of chemotherapy (n=65)**
Median age (range), years	63 (34–86)	62 (34–86)	60 (34–78)	61 (34–78)	66 (34–86)	64 (34–86)
Female, n (%)	122 (62)	104 (65)	67 (61)	56 (64)	51 (65)	45 (69)
Race, n (%)						
White	77 (39)	63 (39)	48 (44)	40 (45)	27 (34)	22 (34)
Asian	105 (54)	87 (54)	54 (50)	43 (49)	45 (57)	38 (58)
Other	13 (7)	10 (6)	6 (6)	5 (6)	7 (9)	5 (8)
ECOG PS, n (%)						
0	68 (35)	57 (36)	41 (38)	35 (40)	26 (33)	21 (32)
1	124 (63)	99 (62)	66 (61)	51 (58)	51 (65)	42 (65)
Brain metastasis at enrolment, n (%)						
Yes	67 (34)	54 (34)	39 (36)	29 (33)	25 (32)	22 (34)
No	129 (66)	106 (66)	70 (64)	59 (67)	54 (68)	43 (66)

*Including 8 patients with unverified MET status who were excluded from the subgroup analysis.

FISH, fluorescence in situ hybridization; FISH5+, FISH (MET copy number ≥5 and/or MET: CEP7 signal ratio ≥2); FISH10+, FISH (MET copy number ≥10); IHC, immunohistochemistry; IHC50+, 3+ IHC overexpression in ≥50% of tumor cells; IHC90+, 3+ IHC overexpression in ≥90% of tumor cells.

Table S2: Efficacy parameters of SAVANNAH study [65].

**Efficacy indicators**	**All patients; IHC50+ and/or FISH5+ (N=193*)**		**IHC90+ and/or FISH10+ (N=108)**		**Not "IHC90+ and/or FISH10+" (n=77)**	
	**All (n=193)**	**No history of chemotherapy (n=157)**	**All (n=108)**	**No history of chemotherapy (n=87)**	**All (n=77)**	**No history of chemotherapy (n=63)**
ORR, % (95% CI)	32 (26, 39)	33 (26, 41)	49 (39, 59)	52 (41, 63)	9 (4, 18)	10 (4, 20)
PFS						
Event (%)	153 (79)	120 (76)	80 (74)	61 (70)	68 (88)	55 (87)
Median PFS, months (95%CI)	5.3 (4.2, 5.8)	4.5 (4.0, 5.8)	7.1 (5.3, 8.0)	7.2 (4.7, 9.2)	2.8 (2.6, 4.3)	2.8 (1.8, 4.2)
Median DoR, months (95% CI)	8.3 (6.9, 9.7)	9.6 (7.6, 15.3)	9.3 (7.6, 10.6)	9.6 (7.6, 14.9)	6.9 (4.1, 16.9)	7.3 (4.1, NC)
DCR, % (95% CI)	61 (53, 68)	59 (51, 67)	74 (65, 82)	75 (64, 83)	43 (32, 55)	40 (28, 53)

The efficacy evaluation set is defined as: patients with measurable lesions at baseline, receiving study drug treatment and RECIST assessment for ≥2 treatment periods; *Including 8 patients with unverified MET status who were excluded from the subgroup analysis.

CI, confidence interval; DoR, duration of response; FISH, fluorescence in situ hybridization; FISH5+, FISH (MET copy number ≥5 and/or MET:CEP7 signal ratio ≥2); FISH10+, FISH (MET copy number ≥10); IHC, immunohistochemistry; IHC50+, 3+ IHC overexpression in ≥50% of tumor cells; IHC90+, 3+ IHC overexpression in ≥90% of tumor cells; NC, cannot be calculated; ORR, objective response rate; PFS, progression free survival.

Table S3: Safety results of SAVANNAH trial [65].

AE, n (%)	All patients (n=196)
Any AE	195 (99)
Any study treatment related AE	164 (84)
CTCAE grade ≥3	88 (45)
Study treatment related AE with CTCAE grade ≥3	39 (20)
Any SAE*	56 (29)
Any treatment related SAE	13 (7)
Any AE resulting in death	13 (7)
Any AE leading to discontinuation of study treatment - savatinib	26 (13)
Any AE leading to discontinuation of study treatment - osimertinib	21 (11)
Any ≥ grade 3 AE	88 (45)
Pulmonary embolism	9 (5)
Difficulty breathing	8 (4)
Decreased neutrophil count	7 (4)
Pneumonia	7 (4)
Hypoalbuminemia	4 (2)
pleural effusion	4 (2)
Vomit	4 (2)
Anemia	4 (2)

The safety analysis set is defined as: all patients who have received ≥1 study drug treatment; *The only SAEs reported in >1 was vomiting (n=2) and pyrexia (n=2).

AE, adverse events; CTCAE, Common Terminology Criteria for Adverse Events; SAE, serious adverse events.
